# A guide to ERK dynamics, part 2: downstream decoding

**DOI:** 10.1042/BCJ20230277

**Published:** 2023-12-01

**Authors:** Abhineet Ram, Devan Murphy, Nicholaus DeCuzzi, Madhura Patankar, Jason Hu, Michael Pargett, John G. Albeck

**Affiliations:** Department of Molecular and Cellular Biology, University of California, Davis, CA, U.S.A.

**Keywords:** cell proliferation, eukaryotic gene expression, extracellular signal-regulated kinases, gene regulatory networks, receptor tyrosine kinases

## Abstract

Signaling by the extracellular signal-regulated kinase (ERK) pathway controls many cellular processes, including cell division, death, and differentiation. In this second installment of a two-part review, we address the question of how the ERK pathway exerts distinct and context-specific effects on multiple processes. We discuss how the dynamics of ERK activity induce selective changes in gene expression programs, with insights from both experiments and computational models. With a focus on single-cell biosensor-based studies, we summarize four major functional modes for ERK signaling in tissues: adjusting the size of cell populations, gradient-based patterning, wave propagation of morphological changes, and diversification of cellular gene expression states. These modes of operation are disrupted in cancer and other related diseases and represent potential targets for therapeutic intervention. By understanding the dynamic mechanisms involved in ERK signaling, there is potential for pharmacological strategies that not only simply inhibit ERK, but also restore functional activity patterns and improve disease outcomes.

## Introduction

The extracellular signal-regulated kinases (ERK) are a subset of the mitogen-activated protein kinases (MAPKs) that regulate many aspects of cellular physiology, including cell growth, proliferation, differentiation, and death. At the organismal level, the ERK family plays an essential role in tissue development, homeostasis, and cancer. Since the identification of the pathway's core components, which are found in all eukaryotes, it has served as a paradigm for how signal transduction pathways convey information from the cell surface to the nucleus to make cell fate decisions [1]. The ability of ERKs to control multiple cellular effects raises the fundamental question of how specific cellular outcomes are determined in a given context [2]. Two basic types of explanation can be proposed. First, unique cellular responses could be determined by contextual cues, such as the coincidence of ERK activation with other signaling activities or cell type-specific gene regulation [3]. Alternatively, changes in the amplitude, duration, or timing of ERK activity — collectively referred to as ‘dynamics' — could specify distinct downstream behaviors. Both of these modes contribute to cellular regulation [2,4], but dynamics-based encoding poses a unique challenge for cell biology [5], in part because standard methods are not well suited to study this type of information processing.

In this review, we focus specifically on the dynamic modes of ERK signaling. We explore the concept that information about the identity and strength of extracellular stimuli is carried (or encoded) not only in the identity of the intracellular molecules activated but in the specific timing of their activation and deactivation [6,7]. In the past 10 years, long-standing challenges in rigorously evaluating the role of dynamics have become tractable through new tools including optogenetics, biosensors, microfluidics, and computational models. These developments have revealed a surprisingly elaborate array of dynamic behaviors of the ERK pathway, which may act as a ‘code' to specify cellular behaviors. In a companion review, we survey the experimental measurement of dynamic ERK activity and mechanistic models of the operation of this ubiquitous pathway. Here, we explore the concept that specific patterns of ERK activity define its downstream effects on gene expression and tissue behavior. These processes effectively ‘decode’ or interpret the information that is encoded in the dynamic activity pattern.

This review focuses on ERK1 (also known as MAPK3) and ERK2 (MAPK1), which have very similar functions in controlling cellular phenotypes. ERK1/2 have been the most studied family members in terms of dynamic activation, and we collectively refer to ERK1/2 as ERK throughout this review. However, other paralogs, which include ERK3, ERK4, and ERK5 also regulate overlapping cellular processes, albeit with significant differences in their specific activities. Very little is known about the dynamic activity of the ERK3/4/5 paralogs and thus we omit them from our focus here.

The concept of temporal coding is already well appreciated in neural circuits, where the timing and frequency of action potentials can transmit specific information between neurons. Compared with action potentials, signaling pathway activity is typically less quantized into discrete peaks, and it usually occurs on a slower time scale (minutes, rather than milliseconds). Nonetheless, it is now established that many signaling pathways show highly dynamic activity, featuring pulses that occur repeatedly on a time scale relevant to pathway function [8,9]. Beyond the ERK pathway, there are several examples of signal transduction pathways in which dynamic activation is prominent, including NF-κB [10,11], p53 [12,13], and Msn2 [14]. There is already an expansive field investigating communication via intracellular calcium dynamics, which have been accessible at the single-cell level for decades through fluorescent dyes [15–17]. Elegant work in each of these systems has established that downstream gene expression patterns are indeed strongly influenced by the temporal pattern of pathway activity [17–19]. In the Msn2 pathway in particular, a careful quantitative analysis of the pathway's temporal coding capacity [20,21] has demonstrated that four distinct expression programs can be determined by different input patterns of Msn2 activation [22].

These findings establish the importance of dynamic encoding in the ERK pathway, which influences many cell fate decisions. Because these cell fates ultimately control tissue homeostasis and repair, the physiological functions of the ERK pathway — to shape tissues during development or regeneration — depend on its temporal activation profile. Furthermore, the dysfunction of the pathway in diseases such as cancer is closely tied to changes in its temporal activity profile [23,24]. In the sections that follow, we explore how the dynamic regulation of ERK defines its downstream effects on gene expression and tissue behavior, with implications for its dysfunction and drug responses in disease.

## Effects of ERK dynamics on gene expression

The cellular effects of ERK dynamics are exerted largely through the regulation of gene expression. ERK has over 1000 identified target genes [25], many of which are themselves involved in transcriptional regulation, allowing ERK to exert widespread influence on the expressed genome. ERK activity can stimulate cell proliferation, differentiation, metabolism, and drug resistance, by modulating the expression of target genes including *CCND1* (encoding Cyclin D1), *FOS* (FOS), *MYC* (MYC), and *FOSL1* (FRA-1). ERK target genes have been categorized into rapidly responding immediate-early genes (IEGs), immediate-late genes (ILGs), and delayed early genes (DEGs) based on their timing of expression following ERK activation [26,27]. Here, we discuss how ERK activity dynamics can lead to differential or selective expression of these genes, which provides a mechanism for the induction of distinct cellular processes.

**Figure 1.**
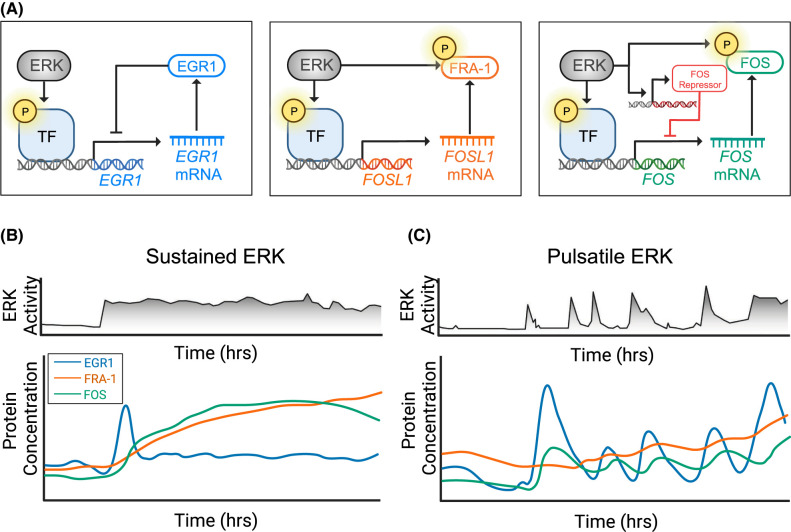
Differential gene expression responses to ERK signaling. (**A**) Differential regulation of *EGR1*, *FOSL1*, and *FOS* expression. In each case, ERK phosphorylates a transcription factor (TF) that binds to the gene promoter. Left: EGR1 protein binds to its own gene promoter, inhibiting further transcription. Middle: ERK initiates *FOSL1* transcription and also stabilizes FRA-1 protein. Right: ERK initiates *FOS* expression, and stabilizes FOS protein through phosphorylation; sustained ERK activation also apparently induces a *FOS* transcriptional repressor that has not been definitively identified [36]. (**B**) Sustained signaling generates high concentrations of FRA-1 and FOS protein. Subsequently, the *FOS* repressor inhibits further expression of FOS. After the initial peak of EGR1 concentration, EGR1 represses its own transcription (auto-inhibition), thus returning to an equilibrium with low baseline expression. (**C**) Pulsatile signaling generates short bursts of EGR1 accumulation. Since *EGR1* transcription is brief and the protein is unstable, auto-inhibition does not persist. This allows EGR1 levels to track closely with ERK activity pulses. Conversely, pulsatile signaling weakly induces FRA-1 and FOS expression because sustained signaling is required for protein stabilization. Figure adapted in part from [24].

ERK activity modulates gene expression at multiple levels. First, ERK directly phosphorylates transcription factors, including ETS family members such as Elk-1, to induce allosteric changes that increase DNA binding and increase transcriptional activity [28,29]. Once mRNA is produced, prolonged ERK activity increases mRNA half-life for certain genes, including ILGs (*EGFR, DUSP6*), but not early (*EGR1, FOS)* or mid (*PHLDA1)* response genes [30]. For many of these genes, the synthesized proteins can also be phosphorylated by ERK to protect them from degradation [31]. Such stabilizing phosphorylations are present in FRA-1, FOS, and other AP-1 family members (Figure 1A) [31,32]. Furthermore, ERK activity regulates chromatin modifiers such as EZH2 and RNA splicing factors such as DAZAP1, which can together modulate the transcriptional productivity of many loci [33]. As we discuss below, these overlapping mechanisms enable the construction of gene regulatory circuits that are sensitive to different spatial and temporal patterns of ERK activity.

### Persistence detection in ERK target genes

The parallel regulation of multiple steps in the gene expression process by ERK creates feedforward regulatory motifs [30]. In principle, this configuration can make target gene expression sensitive to the duration of ERK activity [34,35]. Because stimulation of transcription and protein stabilization both require active ERK but are separated in time, short periods of ERK activity can be unproductive because they stimulate RNA production but do not persist for long enough to phosphorylate and stabilize the newly translated protein. Conversely, persistent ERK activity permits both gene transcription and protein stabilization. Mathematical modeling of this feedforward motif confirms that it can in fact act as a ‘persistence detector' that preferentially responds to longer durations of ERK activity, allowing IEGs such as *FOS* to be selectively induced by growth factors such as Heregulin (HRG) that stimulate prolonged, rather than transient ERK activity [36].

Additional modeling analysis has added depth to this concept, demonstrating that persistence detection depends critically on the kinetic parameters of RNA induction and degradation [26,37]. Effective persistence detection by a gene — whether an IEG, ILG, or DEG — requires that mRNA and protein production be very low in the unstimulated state because any pre-existing pool of protein can be directly phosphorylated and stabilized by ERK, bypassing the feedforward requirement for ERK duration. Consequently, strong persistence detection can be difficult to achieve. Analysis of the ILG *FOSL1* revealed that its product, FRA-1, has a significant basal rate of production and does not require a threshold duration of ERK activity [37]. Instead, FRA-1 production integrates total ERK activity over time, regardless of its duration (Figure 1B). Thus, ERK target genes vary in their capacity for persistence detection and in their responses to ERK activity dynamics [26,37].

In contrast with *FOSL1*, the IEGs *FOS,* and *EGR1* are more sensitive to short-term changes in ERK activity but less responsive to long-term ERK activity (Figure 1C). The mRNAs of these IEGs increase within minutes following ERK activation, which is made possible by rapid mRNA turnover [26]. Because of their short mRNA half-lives, these genes have a higher transcription rate, quickly reach high mRNA levels, and rapidly decay in the absence of ERK activity. At the protein level, FOS and FRA-1 accumulate preferentially when ERK activity persists for more than 30 min, which has been attributed to the need for ERK to remain active long enough to phosphorylate the newly translated proteins [35,36]. Yet, questions remain about how strict this duration requirement is, as the high basal mRNA expression of these genes would relax the requirement [37,38]. Very few studies have quantitatively assessed the response to ERK activity duration in a way that demonstrates a non-linear selectivity for longer stimulation. Quantitative approaches to this question suggest a more complicated situation, as discussed in the section below [36,39]. Nonetheless, mRNA half-life appears to be a critical factor overall, as the short half-lives of IEGs in general allow responsiveness to ERK duration while the longer half-lives of ILGs integrate both ERK duration and amplitude. These gene properties, combined with transcriptional control and post-translational modification, allow for differential gene expression in response to ERK dynamics [24].

### Gene expression as a filter for ERK dynamics

While persistence detection implies effective protein expression for any ERK activity period above a threshold duration, many IEGs, including *FOS* and *EGR1*, actually display a more complex behavior. For these genes, the duration of ERK activity must be within a certain window; both longer or shorter ERK pulses induce sub-optimal expression [39]. This behavior is due to negative feedback, which activates upon longer signaling durations and prevents further stimulation of expression (Figure 1A). Re-stimulation of the pathway before the negative feedback shuts off is counter-productive, and thus over an extended time, the frequency of ERK pulses that can effectively stimulate expression is limited to a certain range. In engineering systems, such behavior is referred to as a ‘band-pass' filter because only frequencies within the preferred ‘band’ can transmit information. Such behavior can be difficult to distinguish from persistence detection without careful experimental manipulation of the input stimuli. However, band-pass behavior has been observed for several genes, including *FOS*, using optogenetic activation of ERK to provide ERK pulses at precise input frequencies [39]. *EGR1* has also been implicated as a band-pass detector because its steady-state expression is more strongly stimulated by pulsatile rather than sustained ERK activation [23,24,40]. Carrying this idea further, mathematical modeling has been used to design synthetic genes that selectively respond to pulses of limited duration, rather than sustained ERK activity [41,42]. These gene designs were experimentally tested in cells and confirmed to respond preferentially, as designed, to pulsatile input patterns.

While the ability to engineer dynamics-selective gene expression responses suggests a high level of understanding, other questions still remain. In particular, it has been noted that the probability of responding to a given ERK stimulus varies from cell to cell [41]. Several studies have developed reporter cells in which both ERK activity and the expression of a target gene can be measured, by using CRISPR-based tagging of endogenous genes with fluorescent protein fusions at genomic loci [37,39]. These systems allow continuous monitoring of ERK and its target gene expression in the same cell. Data from these experiments are useful to examine the fidelity and precision of the process by which ERK drives the expression of IEGs such as *FOS* or *FOSL1*. A striking degree of variability has been found: ERK activity can only explain ∼35% of the variation in *FOSL1* protein expression in single cells [37]. Thus, ERK activity can be strictly required for IEG expression but paradoxically shows a low correlation at the single-cell level. This observation can best be explained by the existence of unmeasured factors that also impact expression, such as epigenetic regulation of gene loci, or metabolic changes that impact transcription or translation rates. Another explanation is that target gene expression may only respond to ERK stimulation in a subpopulation of cells, perhaps due to chromatin modification [43]. In general, the effect of ERK on gene expression at single-cell resolution is not as clear as classical bulk measurements suggest. Future studies will require careful analysis of how ERK activation affects the distribution of its target gene expression within a cell population. Furthermore, there is yet to be a comprehensive quantification of the effects of ERK dynamics on the transcriptional and translational response of multiple ERK target genes in an individual cell, which would reveal whether ERK target genes tend to correlate, or operate independently.

### ERK dynamics in the nucleus vs. cytoplasm as a mechanism for directing cell fate

Subcellular compartmentalization provides another mechanism to generate distinct downstream responses to ERK. The cytoplasmic vs. nuclear distribution of active ERK is guided by differentially localized phosphatases, including the dual-specificity phosphatases (DUSPs), and via anchoring proteins such as PEA-15 [44–47]. The resulting differences in localization can influence cellular behavior in a context-dependent manner. In PC-12 cells, the nuclear import of ERK is important for neuronal differentiation [48–50], while in myoblasts, cytosolic ERK sequestration promotes differentiation during myogenesis [51]. In other systems, nuclear ERK activity promotes proliferation [52] or limits senescence [53]. Therefore, the temporal dynamics of ERK activity are further diversified by protein–protein interactions and complexes that shape its spatial activity, with complex implications for cell fate.

Segregation of ERK activity between the cytosol and the nucleus provides a mechanism for distinct gene expression responses to different ligands. In MCF-7 cells, either epidermal growth factor (EGF) or HRG can induce transient nuclear ERK activity; however, only HRG causes a sustained cytoplasmic ERK response. This sustained cytoplasmic activity stabilizes FOS protein in HRG-treated cells [36]. The transience of nuclear ERK activity in this system is largely due to a negative feedback loop in which ERK activity induces *DUSP4* transcription, which targets nuclear ERK activity, while cytosolic p-ERK remains constant [39]. Conversely, in situations where ERK is primarily active in the nucleus, it would be expected to preferentially phosphorylate nuclear targets such as MYC, JUN, and ETS, relative to cytosolic targets such as RSK1, PDE4, or BIM. The complex dynamic relationship of nuclear and cytoplasmic ERK activity has not yet been fully modeled in most systems, and more studies are needed to understand how different transcriptional programmes can arise from compartmental control of ERK.

## ERK dynamics in tissue regulation

**Figure 2.**
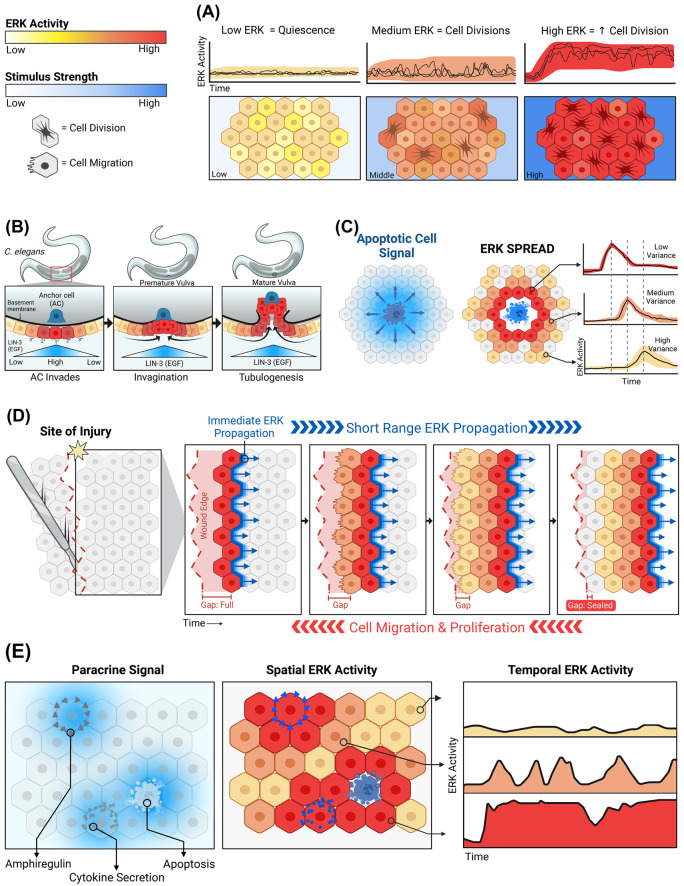
Functions of ERK dynamics during development and tissue homeostasis. (**A**) ERK activity increases the probability of cell division. When exposed to different EGF concentrations (bottom panel, blue), single-cell ERK activity within a population varies from cell-to-cell (cell colors), increasing the average rate of proliferation [59,107]. (**B**) Spatially restricted ERK activity indicates cellular position during development. Within the *C. elegans* gonad, the anchor cell (AC, blue) is the point source for EGF ligand secretion, creating a gradient beneath the basement membrane (blue triangles below cells). Cells closest to the anchor cell receive the most stimulation and thus have the highest ERK activity (individual cell colors, red to yellow), inducing differential cell differentiation programs to create the mature vulva [80,81]. (**C**) Secretion of EGFR ligands from apoptotic cells creates a radial gradient around the dying cell. Cells closest to the center (red) have high ERK activity, which conveys a survival advantage, while cells farther from the dying cell receive a lower concentration of ligands (blue halo in left panel) resulting in lower and delayed ERK activity. Individual ERK activity traces are shown to the right. Figure adapted from Gagliardi et al. [87]. (**D**) During tissue injury, ERK activity provides both directional and migratory signals. Initially, cells adjacent to the injury have high ERK activity (first row, red cells) and secrete a short-range ERK stimulus gradient that activates its neighbors (abutting gradient, blue). Once the neighbors are activated, the ERK activity in the previous cell layer decays, ultimately creating a wave of ERK activity (second through fourth panel). ERK activation waves repeatedly propagate from the site of injury. This directs cells to migrate toward the site of injury (red arrows below) resulting in gap closure and wound healing [55,93,94]. (**E**) Multiple sources of ERK activation generate sporadic spatiotemporal ERK activation. Cells that actively shed EGFR ligands create transient, stimulatory microenvironments (blue gradients), in contrast with (**A**) which depicts uniform stimuli. These dynamic and overlapping microenvironments create varying temporal patterns of ERK activity, generating diverse gene expression profiles within the population [24].

Gene knockout studies in roundworms, fruit flies, zebrafish, and mice have established the genetic requirement for ERK in many developmental and physiological systems. However, knockouts reveal little about the timing and dynamics of ERK activation needed to control tissue function. *In vivo* experiments with live-cell reporters demonstrate transient, localized ERK activation involved during both development and adult tissue homeostasis and wound healing [54,55]. Recent work with optogenetic tools has begun to address this question by making it possible to activate ERK in custom spatiotemporal patterns in a Drosophila embryo [56,57]. Together, these tools are making possible new models of precisely when and where ERK activity is required during development. As new data emerge, we can distinguish four main functional modes for ERK activity dynamics within tissues (Figure 2).

### Setting cell fate probabilities by integrated signal strength or duration

The most basic function of ERK within tissues is as a rheostat for mitogenic activity, where ERK activity controls the progression of the cell cycle (Figure 2A). Canonically, ERK primarily regulates the transition from G1 to S-phase in the cell cycle by inducing transcription of Cyclin D genes (*CCND1/2/3*). The average frequency of entry to S-phase can be modulated by increasing or decreasing ERK activity through growth factors, pharmacological compounds, or cell density [4,58,59]. Cyclin D enables CDK4/6 activity, promoting Rb phosphorylation and a concerted switch in kinase activities and gene expression that commits cells to enter S-phase [60–62]. However, an important question remains to be fully answered, as to which features of ERK activation are essential for activating this transition. Earlier studies suggest that ERK activity timing is crucial during discrete time windows, in cells re-entering the cell cycle after serum starvation [7,63]. However, in cells that are continuously cycling, the probability of S-phase entry for a newly divided cell is proportional to ERK activity throughout the cell cycle of the mother cell [59,64]. A further surprise revealed by this quantitative single-cell analysis is that ERK activity directs the rate of cell cycle entry by setting an overall translational capacity of the cell, and not simply by inducing Cyclin D expression [59]. Yet, an important caveat in many *in vitro* studies of cell cycle regulation is that they employ cells exposed to high levels of growth factor or serum, or cells that are starved and then restimulated with a large bolus of serum or growth factors. These conditions drive rapid cell cycle times (<20 h) and abrupt transitions in metabolism that are unusual within most adult tissues, where cells experience intermittent stimulation by low concentrations of growth factors and cell cycle durations range from days to weeks.

Further complicating ERK's role as a cell cycle driver, high levels of ERK activity can induce cell cycle arrest or senescence, especially when driven by oncogenes [65,66]. In an *in vivo* RAS-driven mammary tumor model, immediate induction of high RAS/ERK activity induced senescence rather than tumorigenesis, and effective tumor growth was only stimulated if RAS activation was initiated at low levels [67]. Conversely, human fibroblast cultures showed very low ERK activity linked to senescence [53]. In both stem cells and non-tumor cell line modes, pulsatile activity is linked to cycle progression whereas sustained ERK activation correlates with cell cycle arrest [68,69]. While these results are context-dependent, a compelling model suggests ERK is a non-monotonic driver of cell fates like proliferation and arrest [66]. Using population and single-cell methods, a recent study confirms that low and high levels of ERK activation induce arrest, whereas intermediate levels lead to maximal proliferation [70].

A similarly complicated situation is found in the regulation of cell death by ERK. Apoptosis in both normal and cancer cells occurs via the release of mitochondrial pro-apoptotic factors including cytochrome *c* to the cytoplasm, which promotes activation of initiator and effector caspases. While ERK is often regarded as a pro-survival signal that suppresses BIM to prevent cytochrome *c* release [71–73], other studies have demonstrated a pro-apoptotic role in inducing caspase-8 activation which stimulates cytochrome *c* release mediated by Protein Kinase B (AKT) activation [74,75]. Paradoxically, both MEK inhibitors and active forms of MEK/ERK/RAF/RAS pathway proteins have been associated with apoptosis induction [47,76]. A recent study used an improved ERK biosensor to clarify the role of ERK in apoptosis and necroptosis in murine fibrosarcoma cells [77]. In response to cell death-inducing ligands, cells demonstrated strong ERK signaling, including an increase in amplitude and duration during the early stages of apoptosis compared with necroptosis. During later stages of cell death, the signaling characteristics of apoptosis and necroptosis were similar, except that early apoptotic cells displayed higher amplitudes [77]. Interestingly, ERK inhibition delayed TNF-induced necroptosis while it sensitized cells to hFas ligand-induced apoptosis, bolstering the context-dependent role of ERK as both a pro-death and pro-survival regulator.

In summary, while ERK is a major overall regulator of quiescence, senescence, proliferation, and cell death rates, we still lack a comprehensive model of how its activity decides between these fates. Developing such a model remains challenging due to the difficulty in accurately measuring the intensity of ERK activity, as well as differences between cell types. The context of activity in other pathways also shapes the effects of ERK. Several studies emphasize cooperative interactions between ERK activity and other kinases, such as PI3K/AKT to enter into S-phase, or JNK to initiate DNA damage-induced-senescence [4,78,79]. Given the above examples, a comprehensive model will need to account for cell type and the context of each cell fate.

### Providing a spatial cue for patterning and maintaining tissues

In many of its physiological roles, ERK activity is localized to certain cells and serves as a spatial indicator. A classic example of this function is the differentiation of vulval precursor cells (VPCs) in *C. elegans* (Figure 2B). Development of this tissue is regulated by each cell's proximity to the EGF-releasing anchor cell (AC). Cells closest to the AC (i.e. VPC P6.p) show an increased frequency of ERK pulses throughout development compared with VPCs that are farther away [80,81]. Critically, changes in the amount of EGF produced by the AC alter the gradient and prevent normal vulval development [80]. Similar examples can be found in the *Drosophila* embryo where ERK gradients play a role in driving cell fates [82–84]. Here, one hour of ERK signaling is required to drive gut-endoderm-like gene expression, while a 30 min pulse initiates an ectodermal neuroblast cell fate [57]. The *Drosophila* embryo is less sensitive to the dose and more perceptive to the spatial distribution and timing of ERK activation [56]. In mouse blastocysts, spatially graded ERK pulses differentiate polar versus mural lineages, and pluripotent blastocysts exhibit opposing levels of basal ERK activity compared with primitive endoderm [85].

Localized ERK signals can also be found in adult organisms, where they help to maintain tissue homeostasis. Local bursts of activity, which were termed SPREADs (for Spatial propagation of ERK activity distributions), have been observed in live mice [55]. Similar patterns are also found in organotypic cultures of epithelial cells, where SPREADs occur preferentially in the outer layer of cells and reduce their propensity for apoptosis, relative to inner cells [86]. In monolayer cultures, apoptotic cells trigger radially propagating waves of ERK pulses, which prevent apoptosis in the surrounding cells (Figure 2C) [87]. By preventing large numbers of adjacent cell deaths, this positional activation of ERK helps to maintain the integrity of the epithelium [54]. In contrast, tissue compaction during *Drosophila* pupal notum development leads to down-regulation of ERK signaling; this local suppression of ERK allows the expression of pro-apoptotic genes, driving excess cell elimination to maintain tissue homeostasis [88]. Altogether, many examples confirm that the localized activation of ERK allows for cells within the correct sub-regions of tissue to adopt fates that allow for appropriate development.

### Driving morphogenesis via wave-like activation

Spatially coordinated, wave-like activity of ERK plays a specialized role in rapid structural changes needed for tissue morphogenesis. ERK regulates cell motility on the scale of hours [89,90], for example in 3D mammary epithelial cell cultures [87] and immune cells [91,92]. Elevated ERK activity can also drive collective migration, a phenomenon crucial for tissue morphogenesis and wound repair (Figure 2D) [68,93,94]. In one example, cell–cell relay of ERK activity across contracting tissues drives invagination in the *Drosophila* tracheal placode. In this process, cells are activated sequentially, with positive feedback from EGFR to the cytoskeletal regulator Rho acting as a switch to cooperatively trigger high ERK activity that is then relayed to the next cell [95]. In another example, spatiotemporal patterns of ERK activity drive base-to-apex multicellular flow in the developing murine inner ear, creating helical collective cell movement that mediates the spiral morphogenesis of the inner ear [96]. This type of tissue behavior is thought to be a product of a mechanical feedback loop where cell extension triggers ERK activation subsequently leading to cell contraction and pulling neighbor cells into an elongated and ERK-activated form [97]. Similarly, zebrafish scale regeneration is triggered by oscillating waves of ERK activity through the regenerating tissue. The wave-like activation is important for their optimal growth as sustained, non-wave-like ERK pattern impairs regeneration times [98]. These examples demonstrate the importance of wave-like ERK signaling in regulating cell motility and tissue morphology.

### Diversification of cell states

Cell fate decisions are often thought of as deterministic responses to a given stimulus, but emerging lines of evidence point to a more probabilistic view. In many of the examples above, the cell fates driven by ERK occur only in a subset of stimulated cells. Yet, this heterogeneity may often be an advantage in physiological tissues. Diversification of cellular behaviors allows a population of cells to adjust its responses over a wider range of stimuli strength and to behave more robustly [99,100]. An interesting possibility is that ERK dynamics may actively facilitate such cellular heterogeneity. The properties of ERK-mediated signaling and gene induction make it well suited to increase the diversity of gene expression within a cell population (Figure 2E) [24]. In comparison with other signaling pathways examined via live-cell imaging, ERK activation is especially sensitive to local environmental stimuli [101], and it can amplify small changes in local growth factor ligands into high dynamic range pulses [37]. As noted above, these pulses trigger disparate patterns of IEG expression, and because IEGs are transcriptional regulators themselves, this variation can translate into diversified expression profiles across the entire genome [24]. Other data corroborate this idea; for instance, during developmental plasticity, autocrine ligands may provide deciding ability to cells by adjusting their fate in response to changing environmental signals [102]. Reversible regulation of transcriptional enhancer activity by ERK may explain this concept: sustained ERK activity induces AP-1 proteins to bind DNA and displace pluripotency transcription factors from genes such as Nanog while fluctuating ERK activity can allow for continued expression of pluripotency network factors [103]. In this context, sporadic ERK signaling contributes to plasticity, allowing cells to maintain multi-fate potential.

Model developmental systems corroborate the role of ERK signaling in modulating expression heterogeneity. In the developing *Drosophila* eye, the transcription factor Yan is regulated by EGFR activity and serves to inhibit differentiation. Variability in Yan expression is important for cellular transitions during photoreceptor cell differentiation, and EGF signals from the cellular microenvironment regulate the duration of this noisy expression period [104]. EGFR signaling appears to be important mainly in directing cells out of the transition state into their respective differentiation paths [104]. In the *C. elegans* vulval system, variability of cell fate induction can have deleterious morphological effects that reduce organismal fitness. Careful quantitative experiments have delimited the range of variation in EGF expression that is compatible with wild-type morphology; expression of EGF above or below this range results in variable cell fates and mutant phenotypes [80]. While ERK signaling has been observed to be variable even within the wild-type range [81], the potential phenotypic outcome of this variation is suppressed by Notch signaling [80]. Thus, EGF–ERK signaling can be an enhancer of cellular heterogeneity in multiple systems, with either functional or deleterious effects, depending on the system.

## ERK dynamics in disease, therapeutics, and pharmacology

The gene expression changes and cellular phenotypes controlled by ERK are central to tissue regeneration, cancer progression, and drug resistance. Sporadic mutations in EGFR, RAS, or RAF that drive ERK activity are strongly associated with cancer development. Inborn mutations in pathway genes cause RASopathies, a family of syndromes that share overlapping developmental abnormalities [105]. Yet, how ERK activity dynamics are altered in either cancer or RASopathy mutant cells has only recently begun to be examined. Traditional methods such as immunoblots fail to distinguish differences in amplitude, intensity, or duration of ERK activity that occur at the single-cell level [100], which may explain why bulk measurements of ERK phosphorylation do not correlate with phenotypes in some models, such as RAS-driven tumor induction [106]. Furthermore, pharmacological pathway inhibitors can differentially alter patterns of ERK activity, even while appearing to produce similar degrees of suppression at the population level [107]. Distinguishing single-cell behaviors is therefore critical in diseases such as cancer where treatment-resistant subpopulations can exert a disproportionately large effect on disease progression [108,109]. Understanding how ERK activity dynamics are altered in diseases, and how they can be modulated for therapeutic benefit, remain important frontiers.

### Differences in ERK activity dynamics in cancer and related diseases

**Figure 3.**
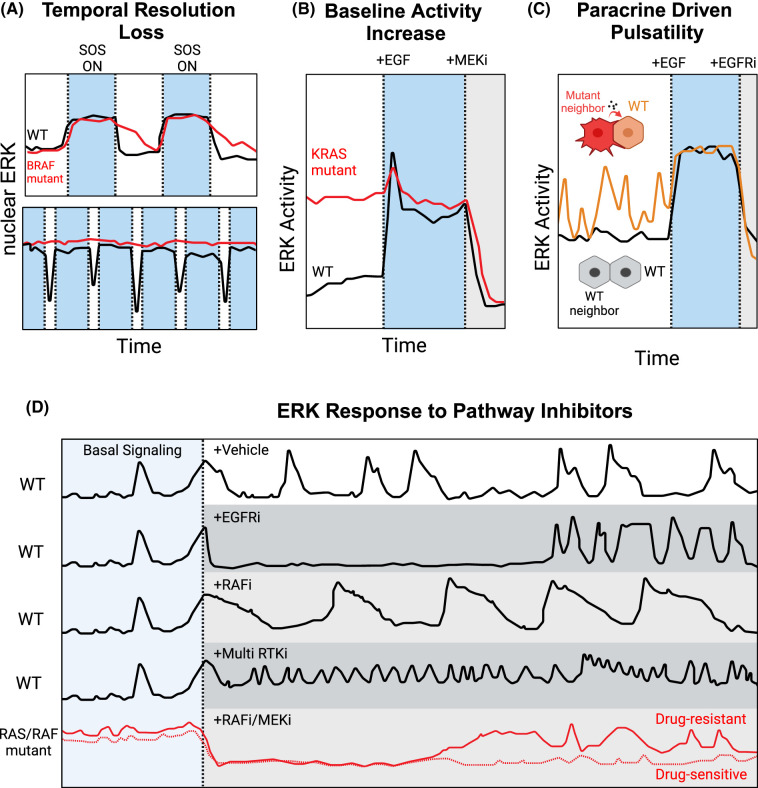
Alterations in ERK dynamics as a result of oncogenic mutations and inhibitors. (**A**) In cells with wild type (WT) pathway genes, ERK undergoes fast activation and deactivation in response to optogenetic stimulation of SOS. BRAF G491A mutation leads to slower deactivation time (top). When stimuli are frequent, ERK responses are elongated into sustained activity (bottom), leading to aberrant transcriptional regulation of target genes [23]. (**B**) Mutations in KRAS drive increased baseline ERK activity. Maximum activity levels upon stimulation remain similar [68,110]. (**C**) Malignant cells release paracrine signals that stimulate sporadic and pulsatile ERK activity in neighboring wild-type cells [24,68]. (**D**) Modulation of ERK dynamics by pharmacological inhibitors. In wild-type cells, the pattern of normal pulsatile signaling (typically resulting from paracrine stimuli [55]) can be altered by the addition of various pharmacological inhibitors (dashed vertical line). EGFR inhibition (EGFRi) quickly abrogates ERK activation, but pulsatile signaling re-emerges within several hours [37]. RAF inhibitors (RAFi) can generate low-frequency ERK pulses [126], potentially as a result of paradoxical activation of RAF. Non-EGFR receptor tyrosine kinase inhibitors(RTKi) result in high-frequency pulses of ERK [126]. In cells carrying oncogenic RAS or RAF mutations, baseline ERK signaling is elevated and inhibition of RAF or MEK (RAFi/MEKi) suppresses ERK signaling initially. A rebound of ERK activity occurs in a subpopulation of cells (solid red line) within several hours, while other cells remain sensitive (dashed red line) [109,128]. All curves shown are approximated depictions of experimental data.

Recent work has begun to examine the distinction between ERK dynamics in normal and cancer cells. This question is complicated by the fact that cancer cells contain hundreds of mutations that can affect pathway function, some of which directly alter the function of the pathway proteins, and others that exert indirect effects by changing contextual factors such as gene expression or cell shape. To simplify the comparison of RAS/ERK signaling properties between cancer cells, Bugaj et al. [23] used an elegant strategy of activating RAS optogenetically at the level of RAS and simultaneously monitoring ERK activation via the nuclear translocation of FP-tagged ERK. This study revealed that tumor cells carrying RAS pathway mutations have reduced temporal resolution in transmitting input light stimuli to the output of ERK activity (Figure 3A). In particular, cells with RAF mutations had a slower off-rate when the input was deactivated, preventing them from distinguishing sequential pulses of input. This loss of fidelity impacted gene expression, as EGR1 showed lower expression levels when pulsatile input signals were blurred at the level of ERK in mutant cells, while Cyclin D1 and c-Jun expression increased, due to the extended active period of ERK [23].

Impaired resolution of stimuli is also found in other examples of RAS/ERK pathway mutations. When mutant RAS isoforms were compared with wild-type RAS within an isogenic ‘RAS-less' background, the major change in ERK dynamics driven by RAS mutants was a higher baseline activity that reduced the amplitude of ERK activity upon growth factor stimulation (Figure 3B) [110]. Additionally, a series of MEK mutants found in RASopathies were analyzed in embryonic *Drosophila* and zebrafish models [111,112]. While these disease-associated mutants varied in whether they increased or decreased ERK activity, a shared feature was a reduction in the dynamic range of ERK activation gradients within each embryo. Finally, in a structural study of mutant EGFR forms found in glioblastoma, these variants showed a reduced difference in their response curves to strong and weak affinity ligands [113]. Together these studies suggest that a general feature of RAS/ERK pathway mutations is the limitation of cells’ ability to respond to time-varying input with distinct amplitudes and durations of ERK activity.

Interestingly, computational models revealed that although the dynamic range of ERK signaling is reduced in RAS mutant cells, it is still much greater than would be expected based on the known biochemical properties of RAS mutants [110]. This observation suggests that the RAS/ERK pathway resists changes in its dynamic range of activity, potentially through feedforward regulation of phosphatases. Theoretical work on signaling pathways has shown that dynamic range is a key parameter for signaling pathways that are made robust by certain pathway structures [114,115]. Thus, the balance between normal and disease-associated signaling may also be determined by the impact of mutations on the pathway's dynamic range. A larger dynamic output range is likely important for transmitting different levels of an input signal, and mutations that decrease this range would have reduced fidelity. This emerging concept pairs well with the idea discussed in the preceding section: the ability to transmit signals in a way that preserves differences in both timing and magnitude may be needed for normal ERK signaling.

Another feature found in models of disease-associated ERK signaling is increased paracrine signaling, which results in greater spatial or temporal variability between cells in a population. Expression of EGFR, RAS, and RAF mutants in a non-tumor epithelial cell line led to increased amphiregulin (AREG) secretion that activated ERK in neighboring cells and triggered increased proliferation, migration, and cell extrusion activities [68]. Another study examined non-tumorigenic mammary epithelial cells in comparison to malignant cells that were selected *in vitro* and *in vivo* for highly invasive behavior [24]. At the single-cell level, these malignant variants exhibit disordered pulses of ERK activity, which are driven by secreted AREG (Figure 3C). In this model, the paracrine AREG signaling drives temporally dynamic ERK activation in neighboring cells, which as noted in effects of ERK dynamics on gene expression, can increase the variation of cellular transcriptional states.

How do ERK pathway mutations drive malignant cell behavior? Considering the changes in ERK dynamics caused by mutations, and the responses of gene expression to these forms of dynamics (see ‘Effects of ERK dynamics on gene expression'), two possible models arise. In the first model, the higher baseline ERK activity due to slow inactivation would drive increased average expression of genes such as *FOSL1* or *CCND1* that tend to accumulate regardless of the dynamics of ERK activity [37,59]. Aberrant expression of *FOSL1* has been linked to cancer stem cells and clonal selection of resistant phenotypes in cancer [116,117]. In the second model, increased paracrine signaling drives a greater degree of time-varying heterogeneity, which may allow individual cells under stress to evade death by modifying their transcriptional profile. In any given tumor, both models could play a role, at different points in time or space. In addition to the simple paracrine signals modeled by *in vitro* systems, ERK activity in cancer cells is likely affected by a mixture of cell–cell, matrix, and nutrient signals from the complex microenvironment, intermixed with the blurring effects of activating pathway mutations [118].

### Manipulating ERK dynamics pharmacologically

Many ERK pathway inhibitors are now available, including small molecule inhibitors of EGFR and other receptor tyrosine kinases (RTKs), certain RAS variants, RAFs, MEKs, and ERKs [119,120]. The development of these drugs was driven by the hope they would effectively target cancers with RTK, RAS, and RAF mutations. However, the history of clinical usage for these drugs is complex, with some proving effective as combination therapies and approved for clinical use in certain cancers, while other candidates were less successful. One explanation for varied performance of ERK inhibitors is the extensive feedback-driven buffering of ERK activity [121,122], which we discuss in the companion review. Presumably, this feedback-mediated stability of signaling is important for physiological homeostasis, but it adds complexity to identifying effective pharmacological inhibitors. Mathematical models have provided one approach to predicting the sometimes counterintuitive effects of pathway inhibitors and their interactions with different mutant configurations [123–125]. In parallel, the effects of 429 kinase inhibitors and related compounds on ERK dynamics have been examined experimentally [126]. This approach identified three classes of compounds based on ERK activity patterns: EGFR or MEK inhibitors strongly suppressed strong ERK activity, BRAF ‘paradox activators' slowed ERK pulsing, and non-ERBB RTK inhibitors increased pulsatile behavior of the keratinocytes (Figure 3D). Together these findings suggest significant potential for engineering the intended pathway activity dynamics using predictive models and single-cell activity data.

Even with highly specific and potent compounds available, complete and sustained inhibition of the ERK pathway is difficult to achieve, and a small portion of dividing ‘persister' cells remain [107,127]. Live-cell imaging confirms that cells progressing through the cell cycle after BRAF inhibition are not due to mutations, but rather incomplete suppression of the pathway and sporadic re-entry into the cell cycle [109,127]. Interestingly, while both of these studies found up-regulation of ERK targets in the persister cells, Gerosa et al. [109] found a spatial clustering of these cells while Yang et al. [127] did not. This discrepancy may reflect differences in mechanisms of resistance, with receptor reactivation allowing paracrine activity in local clusters, while alternate mechanisms occur more sporadically, such as intracellular stress pathway activation. These different mechanisms of resistance yield distinct ERK activity patterns detectable in live-cell data, which could potentially be utilized to formulate personalized treatment plans [126]. Ultimately, the power of live-cell tools may lie in the ability to profile patient-derived tumor tissue or organoids, capturing patterns of dynamic ERK activity that predict effective treatment regimes [128,129]. A major caveat to these studies is that signaling dynamics in isolated tumor cells alone are not reflective of all the possible origins of resistance. ERK dynamics are influenced by numerous other factors including cell density [58], substrate stiffness [130], cell type [131], and the tumor microenvironment [24]. ERK biosensor studies in mouse models of melanoma show that extracellular matrix from cancer-associated fibroblasts promotes more resistant tumor cells that escape pathway inhibition within 12 h of treatment [108]. Future work must address other mechanisms altering ERK dynamics to overcome the multifactorial nature of resistance.

Cellular heterogeneity further complicates ERK targeting efficacy because each cell or subpopulation within a tumor may have different drug sensitivities. Within melanomas, differentiated melanocytic cells are sensitive to BRAF and ERK inhibitors, while dedifferentiated cells that have lost their lineage markers (SOX10 and MITF) are drug-resistant [132,133]. This cell-to-cell variation can be due to differences in signaling pathway activities. Variation in MAPK activation within a population of cells may lead to differential expression of AP-1 transcription factors, which results in changes in heterogeneity and plasticity. In studies with patient-derived melanomas, transcriptional variation is well correlated with drug resistance [134]. Single cells expressing high levels of EGFR prior to vemurafenib inhibition were more likely to become resistant, and this resistant state included increased AP-1 expression. Corroborating these findings, AP-1 expression levels have been found to determine the diversity of states of BRAF-mutant cell lines upon MAPK inhibition. Specifically, cells with high FOS levels correlate with melanocytic and transitory states, whereas FRA1/2 and JUN levels correlate with undifferentiated cells. Furthermore, knockdown of AP-1 factors leads to changes in differentiation state markers, suggesting that targeting AP-1 factors may render cells more vulnerable to pharmacological treatment [135,136]. Thus, heterogeneity and adaptive tendencies within highly mutated carcinomas continue to be a challenge for cancer therapy.

Modulating the ERK pathway has great potential in treating RASopathies. One of the most prevalent RASopathies is neurofibromatosis type I, which affects individuals with mutations in the *NF1* gene. NF1 normally down-regulates the ERK pathway by inactivating RAS, and individuals carrying a heterozygous loss of function of NF1 allele develop numerous neurofibroma tumors throughout their lives [137]. Plexiform neurofibromas (pNFs) cause morbidity and can progress to malignant peripheral neural sheath tumors (PNSTs). Sustained treatment with the MEK inhibitor selumetinib has shown substantial effectiveness at reducing the growth of pNFs, demonstrating that a sustained reduction in signaling activity over time can counteract the increased ERK activity which primarily arises from NF1 loss [138,139]. Further investigations are needed to determine the potential of similar strategies against different RASopathies, and also to decipher if they harbor distinct forms of ERK activity amplification that require different forms of inhibition.

### Harnessing ERK activation dynamics for stem cells and regeneration

While the primary focus of most therapies in cancer and RASopathies is to inhibit ERK, manipulating ERK dynamics in other ways has utility in regenerative therapies. One emerging tool is the use of stem cells to repair injured tissues. ERK plays a multifaceted role in stem cell pluripotency and cell fate choices, which makes pharmacological targeting of the pathway a potential route for controlling differentiation. ERK activation, typically through Fibroblast Growth Factor (FGF), leads to differentiation of embryonic stem cells (ESCs) [103,140–142]. Cultured ESCs can only be maintained in their naive, fully pluripotent state if ERK activity is strongly suppressed [143]. To maintain their pluripotency, culture media dubbed ‘2i' and ‘3i' have been developed. 2i media includes inhibitors for both MEK and GSK3, with the MEK inhibitor suppressing ERK activity and the GSK3 inhibitor activating the transcription factor beta-catenin, while 3i additionally includes an FGFR inhibitor. Live-cell imaging revealed substantial heterogeneity in ERK activity in ESCs following removal of 2i media, which coincided with loss of expression of the pluripotency factor Nanog over time [144]. Further study of ESCs revealed that they respond with a unique form of ERK activity: periods of high-frequency oscillations that are more regularly spaced and have a much shorter period (∼7 min) than pulses found in epithelial cells [145].

The precise control of stem cell differentiation by ERK is an area of ongoing study. An extensive investigation of biosensor dynamics in hematopoietic stem cells and multipotent progenitor cells revealed that the fate choices of adult stem cells are strongly influenced by ERK signaling [146]. Live-cell imaging of these primary cells revealed differential responsiveness to different cytokines, as well as strong variation in ERK response between cells of the same type. Importantly, the specific pattern of ERK activity in individual cells predicted the future emergence of differentiation markers, suggesting that heterogeneity in ERK dynamics plays a role in setting the proportions of cells of different cell fate. However, the specific effect of ERK activity on gene expression is context-dependent. Deathridge et al. [144] found a surprisingly weak correlation between ERK activity and Nanog expression in ESC differentiation, while a study in blastocyst development found a negative correlation [147]. Therefore, ERK activity in this context likely controls cell fate by biasing gene expression and driving differentiation of cells in a proportional manner [148]. These data provide important insights into engineering signaling patterns in stem cells to control differentiation programs [149,150], with potential applications in treating a wide range of diseases.

Along with stem cell therapies, directly altering ERK signaling within injured tissue may be useful to improve healing. As previously discussed, waves of ERK activity coordinate epithelial migration to close wound gaps [93,94]. Additionally, ERK activity also helps direct the movement of immune cells [91,92] and fibroblasts, which produce the scaffolding that is necessary for reepithelialization [151]. Live-cell imaging in zebrafish also indicates that sustained ERK activity is required for angiogenesis [152]. Transient epithelial-mesenchymal transition (EMT) is thought to be an important part of this process [153,154], whereby ERK facilitates the temporary phenotypic changes that result in loss of cell–cell contacts, increased cell motility, and increased expression of matrix proteins [155–159]. ERK activation leads to epigenetic remodeling and expression of transcription factors that facilitate global gene expression changes [156,160]. Mathematical models based on immunofluorescence signaling data suggest that changes in signaling dynamics play a role in EMT [161]. While the exact forms of ERK dynamics that drive EMT are still unclear, a study by Aikin et al. [68] showed sustained ERK activity via BRAF and MEK mutations induced increased motility and decreased epithelial cell markers, consistent with an EMT-like phenotype. More time-resolved signaling data would greatly contribute to our understanding of wound healing, as it is clear that ERK is necessary for initial wound healing and is maintained during healing by various growth factors released at the site of injury [55,93,98]. Inhibition of ERK activity prolongs wound closure and could result in scar tissue [94,162]. Conversely, ERK also promotes aberrant EMT and fibrosis [163–165]. Resolving the timing and patterns of ERK activity necessary for proper wound healing could therefore allow for rational design of therapies for faster healing with minimal scar tissue [166].

## Future directions for dynamic ERK encoding

The ERK pathway is a powerful and flexible communication system that has been adapted by evolution to perform many biological functions. While early models envisioned deterministic signaling patterns triggered by distinct growth factors that encoded discrete outcomes, single-cell studies have provided a much more nuanced view. In general, the relationship between ERK activation, gene expression, and cell fate is not perfectly correlated in individual cells. Despite the complexity of this connection, the emerging principles discussed in this review help to make sense of the intricate patterns and their biological functions and bring us closer to understanding the ‘code' of ERK dynamics that determines cell behavior. Yet, while these principles help make sense of the existing data, they also argue for increased sophistication in designing experiments and developing models.

One main concept is that while ERK is a strong causal driver of tissue-level cell behavior, its actions at the single-cell level are imprecise. Even for genes primarily regulated by ERK, detailed measurements of a cell's ERK activity provide only limited predictive power for a given gene [37,144]. Thus, the majority of the literature points to an essential interplay between signaling and contextual factors, rather than the dynamics alone. In other words, information is transmitted by ERK dynamics, but its interpretation is contingent on the status of other pathways within each cell. A similar conclusion has been reached in other signaling pathways [167], and thus signaling in general should be viewed not as a way to precisely specify a downstream response within a tissue, but rather as a means to influence the probability of a given cell behavior. While we often study signaling pathways in isolation, each cell — and even each individual gene within the cell — responds to many inputs simultaneously. Viewed from this perspective, each signaling pathway is only a piece of the puzzle in determining cell behavior. Thus, returning to the two overarching models of regulatory encoding — cellular context vs. dynamics — it is clear that both play a significant role in determining cell behavior. A key question for future work will be to understand how many different pathways must be interrogated to predict cell behavior with high confidence. Lessons from the ERK pathway will need to be integrated with similar insights from other pathways such as NF-κB, p53, and cyclin regulation for which similarly extensive quantitative concepts have been developed. New methods will be needed to interrogate additional pathways, such as metabolic regulation, and to monitor multiple pathways simultaneously within each cell.

A second major concept is that the effects of ERK on tissue morphology and homeostasis cannot be understood simply as actions by single cells but rather must be considered as the collective behavior of cell groups. For example, in the case of ERK-stimulated migration, the aggregate wave-like activation of ERK within a cell monolayer drives the directionality of the migration [88]; the ERK activity of any individual cell is insufficient to understand or predict the behavior of this system. Similarly, during scale regeneration or mouse ear development, wave propagation is crucial for proper ring-like growth of the tissue [96,98]. Although ERK is activated at the single-cell level, it is the aggregate cell behaviors that result in changes in morphology [168]. Therefore, assessments of ERK activity need to consider distributions of pathway activity — in time, space, and magnitude — rather than either means or single-cell values, if they are to provide sufficient information to predict and manipulate ERK activity within its physiological context.

A challenge brought to the forefront by these emerging concepts is how spatiotemporal ERK dynamics can be evaluated *in vivo* or in clinically relevant models, such as patient-derived tissues. While live-cell biosensors are becoming standard tools to study ERK dynamics *in vitro*, incorporation of these sensors is not yet practical in clinical settings. However, given that several genes respond selectively to different patterns of ERK activity, we envision a model that allows for inference of ERK dynamics in patients without the use of biosensors. Future models based on the known response characteristics could in principle decode immunohistological or gene expression data from fixed tissue samples to infer the patterns of ERK activity that were present in a tissue in the hours or days before it was collected. Knowledge of this cellular signaling history could provide crucial data to better detect oncogenic signaling activity or predict pharmacologic efficacy.

## Open Access

Open access for this article was enabled by the participation of University of California in an all-inclusive *Read & Publish* agreement with Portland Press and the Biochemical Society under a transformative agreement with UC.

## Abbreviations

AC: anchor cell
AP-1: activator protein 1
AREG: amphiregulin
ATP: adenosine triphosphate
BIM: Bcl-2 interacting mediator of cell death
BRAF: v-raf murine sarcoma viral oncogene homolog B1
CDKs: cyclin dependent kinases
cMyc: cellular myc
cPLA2: phospholipase A2
CRISPR: clustered regularly interspaced short palindromic repeats
Cyclin D: cyclin protein
DAZAP: DAZ-associated protein
DUSPs: dual-specificity phosphatases
EGF: epidermal growth factor
EGFR: epidermal growth factor receptor
EGR1: early growth response 1
EMT: epithelial-mesenchymal transition
ERK: extracellular signal-regulated kinase
ETG: ERK target genes
ETS: erythroblast transformation factors
EZH2: enhancer of zeste homolog 2
FGF: fibroblast growth factor
fMLP: N-formyl-Met-Leu-Phe
FOS: c-Fos proto-oncogene
FRA-1: Fos related antigen 1, encoded by *FOSL1* gene
GSK3: glycogen synthase kinase 3
HRG: Heregulin
IEG: intermediate-early gene
JNK: c-Jun N terminal kinase
Jun: transcription factor
MDCK: Madine darby canine kidney
MEK: mitogen-activated protein kinase kinase
mESC: mouse embryonic stem cells
Msn2: stress-responsive transcriptional activator
mTORC1: mammalian target rapamycin complex 1
MYC: human transcription factor
NF1: neurofibromatosis type 1
NK-kB: nuclear factor kappa B
P53: tumor protein P53
PDE4: phosphodiesterase-4
PHLDA1: Pleckstrin homology-like domain family A member 1
pNFs: Plexiform neurofibromas
PNSTs: peripheral neural sheath tumors
PrE: primitive endoderm
RAF: serine/threonine protein kinase
RAS: rat sarcoma
Rho: Rhodopsin
RSK: ribosomal S6 kinase
SPREADS: spatial propagation of radial ERK activity distribution
TSC: tuberous sclerosis complex
VPC: vascular progenitor cells
